# Factors affecting task shifting/sharing from physicians to pharmacists in cancer chemotherapy in Japan: A qualitative study

**DOI:** 10.1371/journal.pone.0358247

**Published:** 2026-09-15

**Authors:** Keiko Ikuta, Atsushi Yonezawa, Hiroshi Okada, Hanako Naganawa, Mitsuhiro Sugimoto, Yasuaki Ikemi, Mamiko Saigo, Akihiro Manaka, Tomohiro Terada

**Affiliations:** 1 Department of Clinical Pharmacology & Therapeutics, Kyoto University Hospital, Kyoto, Japan; 2 Division of Integrative Clinical Pharmacology, Faculty of Pharmacy, Keio University, Tokyo, Japan; 3 Department of Preventive Medicine, Kyoto University School of Public Health, Kyoto, Japan; 4 Bureau of Health Security and Management, Japan Institute for Health Security, Tokyo, Japan; University for Continuing Education Krems: Universitat fur Weiterbildung Krems, AUSTRIA

## Abstract

**Introduction:**

There is an increasing demand for pharmacists in Japan’s health care system, particularly in cancer chemotherapy. Task shifting/sharing has enabled clinical pharmacists to take a more active role; however, only a few hospitals have adopted this method, suggesting that institutional factors influence its implementation. Therefore, this study aimed to explore the process of implementing task shifting/sharing from physicians to pharmacists and identify the factors of promoting task shifting/sharing in cancer chemotherapy in Japan.

**Methods:**

We conducted semi-structured focus group interviews with hospital pharmacists in management and nonmanagement positions, between February 2023 and May 2024. All interviews were recorded, transcribed, and independently coded by two researchers. A modified grounded theory approach was used, and data collection continued until theoretical saturation was reached.

**Results:**

Data saturation was reached after 10 group interviews and two additional interviews were conducted to ensure the comprehensiveness of the data. Five categories were identified: “Barriers,” “Cultural change,” “Workflow redesign,” “Improving professional knowledge and clinical skills,” and “Success of interprofessional collaborative practices.” Revisions to medical fees and government notifications facilitated task shifting/sharing; however, most hospitals encountered significant barriers. Three key factors—“Cultural change,” “Workflow redesign,” and “Improving professional knowledge and clinical skills”—were essential in overcoming challenges and promoting implementation.

**Conclusions:**

This study highlighted the process of promoting task shifting/sharing in cancer chemotherapy. Most participants perceived that this initiative improved patient outcomes and safety and reduced the workloads of health care professionals. To further promote this initiative, it is crucial to evaluate and publish evidence of its effectiveness. Moreover, sustained support from government bodies and professional organizations is vital for broader implementation.

## Introduction

The World Health Organization (WHO) reported in 2022 that the global health care workforce shortage is expected to continue until 2030 [1]. Pharmacists play an increasingly vital role in health care [2]. Currently, they are integrated into health care teams in several countries and their inclusion improves treatment outcomes [3]. The Collaborative Drug Therapy Management (CDTM) in the United States [4–7], the Pharmacist Independent Prescriber (PIP) model in the United Kingdom [8], and independent prescribing initiatives in Canada and New Zealand [8,9] have had positive effects on patient outcomes. These examples demonstrate the growing involvement of pharmacists in patient care and their role in improving health care quality.

In Japan, the Ministry of Health, Labor and Welfare issued a document titled “Promotion of Task Shift/Share within the Scope of the Current System” [10]. This policy outlines how clinical and administrative duties can be redistributed from physicians to other health care professionals under the current legal framework, in response to severe physician labor shortages and the 2024 overtime‑work regulations. Protocol-based pharmacotherapy management (PBPM) represents a prime example of task shifting/sharing executed as protocol-based co-management between physicians and pharmacists [11]. It was designed as a Japan-specific adaptation to align collaborative care with the country’s legal and clinical frameworks [12]. Unlike the United States’ CDTM and the United Kingdom's PIP model, PBPM operates under stricter institutional and legal constraints in Japan, functioning as a highly standardized, protocol-driven delegation of tasks rather than a broad collaborative practice agreement. Given this background, it is difficult in Japan to completely shift physicians’ duties to other health care professionals; instead, a combination of task shifting and task sharing is employed. However, PBPM has demonstrated substantial utility, including enhancing patient quality of life [13–15] and optimizing the utilization of appropriate testing [16].

Despite its demonstrated effectiveness, only a few hospitals have implemented task shifting/sharing [13–16]. This suggests that barriers to promoting this initiative exist within individual facilities, although the reasons remain unclear. Therefore, we aimed to identify factors that promote the implementation of task shifting/sharing and generate a process model of implementing task shifting/sharing from physicians to pharmacists in cancer chemotherapy in Japan using a qualitative study. This approach is uniquely suited to capture the deep, nuanced insights of health care professionals [17]. The findings of this study may contribute to a deeper understanding of the processes through which task shifting/sharing from physicians to pharmacists can be promoted in cancer chemotherapy.

## Methods

### Study design

This study used semi-structured focus group interviews to better understand the barriers and facilitators of promoting task shifting/sharing among hospital pharmacists. Using the focus group interviews, interactions among group members can reveal multiple aspects of the problem under evaluation. Furthermore, information regarding the individual perspectives of each member within the group is often obtained simultaneously. This report follows the Consolidated Criteria for Reporting Qualitative Research (COREQ) reporting guideline (S1 File) [18].

### Sampling and recruitment

We used the theoretical sampling technique, until theoretical saturation was reached and an explanation was generated for the process or phenomenon under investigation [19,20]. We were keen to capture specialized insights regarding pharmacist duties related to chemotherapy. The eligibility criteria for this study were pharmacists with at least three years of clinical experience who worked at hospitals at the forefront of task shifting/sharing and were involved in chemotherapy-related duties [21]. Initially, we used purposive sampling to recruit participants who met the eligibility criteria. As data collection and analysis proceeded concurrently, tentative categories emerged, suggesting that conditions within both the pharmacy department and the wider hospital influenced the implementation of task shifting/sharing. To clarify the underdeveloped dimensions of some concepts, theoretical sampling was employed. In addition to meeting the initial eligibility criteria, additional participants recruited at this stage were pharmacists working at hospitals that had recently and successfully implemented task shifting/sharing. This targeted sampling allowed a deeper exploration of the process of implementing task shifting/sharing from physicians to pharmacists in cancer chemotherapy. We contacted the participants by email. Although participation was voluntary, not a single person declined. No one else was present besides the participants and researchers.

### Data collection

Two semi-structured focus group interviews were conducted at each site in university and general hospitals between February 1, 2023, and May 31, 2024, following the interview guide (S2 File); one for pharmacists in management positions and one for those in nonmanagement positions. Participants were informed of the study’s purpose and provided written informed consent. Each group was interviewed only once; there were no repeat interviews. KI conducted the interviews under the supervision of AY and HO. The interviews were recorded in the workplace and transcribed verbatim. Saturation was assessed via sample size adequacy, specificity, theoretical background, dialogue quality, and analysis strategy [20]. We evaluated the interview content at the end of the study to ensure that no new information emerged.

### Data analysis

The interview data were analyzed using a modified grounded theory approach [22]. Transcripts were uploaded onto the qualitative data analysis software NVivo 12.0 (QSR International Pty Ltd.) [23]. Data analysis was conducted by KI and AY under the supervision of HO. KI conducted line-by-line coding of the interview transcripts, reading each transcript multiple times and generating initial codes and analytic memos grounded in the data. Using the constant comparative method, KI and AY continuously compared each code and data segment with the rest of the dataset to refine concepts and develop analytical categories [24,25]. Interpretive differences were discussed among KI, AY, and HO through regular meetings, during which emerging categories, relationships, and clinical implications were critically reviewed. This iterative analytic process proceeded concurrently with data collection and theoretical sampling, consistent with grounded theory methodology. In the final analysis phase, we used ongoing memos and constant comparison to examine how different categories were related to each other. Specifically, we focused on how each factor influenced the implementation of task shifting/sharing—either as a barrier or a facilitator. By analyzing these dynamic relationships among the categories, we combined these connections to build our final process model. After 10 interviews, theoretical saturation was reached because no new conceptual categories or properties emerged. However, to ensure the comprehensiveness of the data, two additional interviews were conducted. We did not return the transcripts to participants for comment; therefore, no one provided feedback on the ﬁndings.

### Researcher’s characteristics and reflexivity

To ensure transparency and rigor, we clearly stated the research team’s position and any potential conflicts of interest. The primary interviewer was a pharmacist with a PhD in epidemiological research and less than two years of clinical experience and received training in qualitative methodology from an experienced qualitative researcher. To ensure analytical depth and contextual accuracy, the research team included two senior researchers as supervisors: a pharmaceutical researcher with extensive clinical experience and an expert in qualitative research. The senior researchers actively participated in the research design, ongoing analytic discussions, and data interpretation, offering diverse and complementary perspectives that strengthened the overall rigor of the study. We recognized that our shared professional backgrounds as pharmacists might lead us to hold positive assumptions about expanding the pharmacist’s role. To enhance reflexivity, we kept field notes after each interview and held regular discussions among the research team to reflect on how our perspectives and interactions might influence data collection and interpretation. Pilot interviews were conducted at a facility separate from the study sites under conditions that simulated the main study, primarily to train the interviewer. Furthermore, through these pilot interviews, the team validated the interview guide (S2 File), identified potential biases, and refined the interview questions. In addition, none of the researchers had any prior personal or professional relationships with the participants or participating institutions, ensuring that the interviews were conducted from an independent standpoint. Regarding the expansion of pharmacist-led oncology services and task shifting/sharing, our team had no organizational, financial, or professional stake in promoting this specific model. Reflexivity was maintained throughout the study to ensure transparency and credibility.

### Ethics approval and consent to participate

This study was performed in line with the principles of the Declaration of Helsinki*.* The study protocol was approved by the Kyoto University Hospital Ethics Committee (R3737-1). Each participant was informed of the study’s purpose and provided informed written consent.

## Results

Overall, 31 hospital pharmacists participated in this study. Data saturation was reached after 10 group interviews and two additional interviews were conducted to ensure the comprehensiveness of the data; thus, data from these 12 focus group interviews were analyzed. There were 2–3 participants in each group and each interview lasted approximately 60 min. The baseline characteristics of the participants are summarized in Table 1. The analysis resulted in 17 subcategories from which five major categories emerged (Table 2, S1 Table).

**Table 1:** Characteristics of participating pharmacists in this study (n = 31).

Total, n		Pharmacy manager	Staff pharmacist
	31	15	16
Professional qualifications, n			
Board Certified Oncology Pharmacy Specialist	14	4	10
Board Certified Senior Oncology Pharmacist	7	6	1
Board Certified Pharmacist in Oncology Pharmacy	3	1	2
Accredited Pharmacist of Ambulatory Cancer Chemotherapy	1	0	1
Male, n	18	11	7
Age (years), n			
≥50	7	7	0
40–49	11	7	4
30–39	10	1	9
20–29	3	0	3
Years of service, n			
≥20	14	12	2
10–19	11	3	8
5–9	5	0	5
3–5	1	0	1

**Table 2:** Key categories and subcategories of promoting task shifting/sharing.

Categories	Subcategories
Barriers	Differences in physicians’ acceptance of task shifting/sharing
	Difficulties in clearly distinguishing the job description of pharmacists from that of physicians
	Excessive workload and shortage of human resources
	Skills of pharmacists in charge of pharmacist-led clinics
Cultural change	Fears of dependence on the competence of a particular pharmacist
	Medical fee revisions and notifications from the government
	Recognition of pharmacists’ roles by physicians and other team members
	Behavioral changes from pharmacists’ perspective
	Wider executive support
Workflow redesign	Reorganization of operations
	Expanding employment of pharmacists
	Improvement of hospital infrastructure
Improving professional knowledgeand clinical skills	Training and updating in clinical skills
	Creation of guidelines and procedures for pharmacy operations
Success of interprofessional collaborative practices	Effectiveness of task shifting/sharing
	Importance of assessing the effect of task shifting/sharing

### Barriers

The first category was barriers to the process of promoting task shifting/sharing. Participants identified a range of barriers.

#### Differences in physicians’ acceptance of task shifting/sharing.

A significant barrier was the acceptance of task shifting/sharing among physicians, which differed by medical specialty. For instance, surgeons were more receptive than internal medicine physicians. This discrepancy may exist because surgeons focus on surgery whereas internal medicine physicians focus on drug therapy.

*“There are still some physicians who have not fully embraced the idea, but I feel that the number of those who are reluctant to accept proposals has significantly decreased. However, there may still be a few who remain resistant to change.” (Facility 4, Staff pharmacist)*

*“Surgeons may be more willing to delegate tasks, such as writing prescriptions, to pharmacists. They often believe that their primary roles are performing surgeries and making diagnoses, allowing them to focus on these core competencies.” (Facility 5, Pharmacy manager)*

*“Since internal medicine doctors primarily focus on prescribing medications, delegating this task to pharmacists could impede the development of medical residents. As a result, progress in this area has been slow.” (Facility 1, Pharmacy manager)*

#### Difficulties in clearly distinguishing the job description of pharmacists from that of physicians.

Some participants suggested that the lack of a clear role distinction between pharmacists and physicians, unlike other health care professionals, could be a potential barrier to implementation.

*“Pharmacists often find it challenging to define clear roles and responsibilities when collaborating with physicians or discussing task division among different professions. This ambiguity can lead to uncertainty and hesitation in decision-making.” (Facility 6, Pharmacy manager)*

#### Excessive workload and shortage of human resources.

Across all facilities, participants noted staff shortages that increased pharmacists’ workloads. Participants also reported insufficient spaces for pharmacist-led clinics.

*“The number of pharmacists and available space are indeed significant challenges that serve as initial barriers.” (Facility 4, Pharmacy manager)*

#### Skills of pharmacists in charge of pharmacist-led clinics.

The interviews strongly suggested that a lack of knowledge and experience regarding cancer chemotherapy is a major barrier to task shifting/sharing. While arising from different perspectives, concerns about clinical competence represented a common recognition across all organizational levels. From an administrative and patient safety perspective, managers questioned whether staff possessed sufficient skills, proposing that those conducting pre-consultation interviews or handling delegated prescriptions should hold specific professional certifications. Practicing pharmacists expressed similar concerns about their own clinical preparedness; they reported pressure associated with increased responsibilities, compounded by perceived gaps in their oncology expertise.

*“It can be challenging to adjust duties and placements when selecting individuals with a certain level of experience rather than exclusively younger staff. Additionally, coordinating tasks and responsibilities within the ward can also present difficulties.” (Facility 5, Pharmacy manager)*

*“When it comes to areas where one’s knowledge may be lacking, it can be challenging to input all prescriptions for each patient and ensure that the prescriptions align with the physician’s intentions.” (Facility 5, Staff pharmacist)*

#### Fears of dependence on the competence of a particular pharmacist.

In addition, some pharmacists expressed concern that, to ensure the quality of medical care, limiting pharmacists who conduct pre-consultation interviews and enter proxy prescription orders to those holding certifications related to cancer pharmacotherapy could restrict the number of pharmacists eligible to participate in task shifting/sharing, potentially leading to an excessive concentration of workload on those certified pharmacists.

*“Fortunately, we have several oncology pharmacists, and we operate the outpatient treatment center with a team of two as a form of mentorship for younger staff. However, if one person ends up shouldering all the responsibilities, it could lead to significant burdens, especially if that person suddenly needs time off or is absent. Ultimately, it is crucial to ensure a sufficiently staffed pharmacy department to prevent such situations.” (Facility 3, Staff pharmacist)*

### Cultural change

Three key factors were identified from the interview survey to overcome the barriers to initiating task shifting/sharing. The first was health care professionals’ perceptions. From the interviews, it was indicated that a positive attitude of health care professionals and government officials toward pharmacists’ involvement in cancer chemotherapy is an enabler of task shifting/sharing.

#### Medical fee revisions and notifications from the government.

Several institutions have initiated and expanded task shifting/sharing. From our interviews, this trend may be driven by new reimbursement for pharmacists’ roles and guidance from administrative and academic bodies.

*“It is my understanding that the impetus for the widespread implementation of task shifting/sharing is the government’s mandate to reform physicians’ working conditions.” (Facility 6, Pharmacy manager)*

#### Recognition of pharmacists’ roles by physicians and other team members.

Furthermore, some participants suggested that the consistent performance of pharmacists’ duties led to a perceptual shift among physicians and other health care professionals, ultimately resulting in greater recognition of the pharmacists’ roles.

*“I believe that consistently making such proposals has led to a greater acceptance of pharmacists. Whether it is in the pharmacist outpatient clinic or during rounds, by persistently advocating for these changes, they have become more readily accepted.” (Facility 4, Staff pharmacist)*

#### Behavioral changes from pharmacists’ perspective.

Participants commented on the pharmacists’ perspectives on task shifting/sharing, raising concerns about the increased workload and accountability its implementation would bring. They also highlighted the importance of mutual role understanding among pharmacy staff for its expansion.

*“Since taking on more responsibilities in the pharmacy department inevitably means more work and accountability, it is understandable that pharmacists have not been actively stepping forward to request additional tasks or roles.” (Facility 3, Staff pharmacist)*

*“If we could share responsibilities within the pharmacy department, considering differences in tasks and workload on different days or times, including within the central pharmacy, it would be beneficial if we could better understand each other’s roles.” (Facility 5, Staff pharmacist)*

#### Wider executive support.

Participants suggested that securing the understanding and consent of hospital executives would make initiating and expanding task shifting/sharing more feasible.

*“I believe system modifications require financial investment, so if the hospital is willing to invest in them to the extent necessary, and if pharmacists are genuinely desired in that regard, then it truly indicates progress.” (Facility 3, Staff pharmacist)*

### Workflow redesign

The second key factor for overcoming the barriers was securing physical resources, specifically ensuring sufficient pharmacy department capacity and the availability of necessary equipment.

#### Reorganization of operations.

Reorganizing work processes within the pharmacy department is crucial for mitigating pharmacists’ workloads. Some participants reported that standardizing workloads can increase operational efficiency.

*“I think we could also handle chemotherapy orders ourselves. To pave the way for this, we could work on standardizing dosage criteria and other related factors. By doing so, we could create a situation where pharmacists are empowered to place orders themselves.” (Facility 2, Pharmacy manager)*

*“Standardizing the approach to side effects and proposing its usefulness for younger pharmacists. It serves an educational purpose for them as well.” (Facility 2, Pharmacy manager)*

Sharing information about task shifting/sharing with neighboring pharmacies could improve efficiency and reduce the burden on health care professionals.

*“Since we use a common monitoring instruction manual for side effects across all medical departments, I think there is a sense of shared responsibility or delegation from physicians to pharmacists regarding the explanation of side effects to patients. By using the same shared manual, it becomes easier for pharmacists to provide follow-up between outpatient visits.” (Facility 1, Pharmacy manager)*

Some participants suggested delegating duties to non-pharmacists, enabling pharmacists to focus on more specialized duties.

*“We could consider shifting some of the pharmacist workload to nurses if there are tasks they can handle.” (Facility 1, Pharmacy manager)*

*“It may be necessary to monitor what truly matters and what the physicians’ requests are.” (Facility 1, Pharmacy manager)*

#### Expanding employment of pharmacists.

While process improvements can help, increasing the number of pharmacists is necessary to reduce workloads.

*"In order to implement task shifting/sharing, the first step is to secure the necessary personnel and ensure there is enough capacity.” (Facility 5, Staff pharmacist)*

#### Improvement of hospital infrastructure.

With the increasing trend of task shifting/sharing, pharmacists are likely to have more opportunities for direct patient care. Consequently, establishing pharmacy-led outpatient clinics is essential.

*“At the outpatient treatment center, the timing seemed to be favorable, as it started in 2007 when there were various advancements in cancer care at the national level. Chemotherapy treatments were transitioning from inpatient to outpatient settings. It coincided with the completion of the three wards at the outpatient treatment center. Originally, the current location was planned to be a lookout restaurant, but owing to circumstances, it was hastily converted into the outpatient treatment center. Instead of forcing it into an existing space, we were fortunate to create a new facility, which I think was fortunate in that sense.” (Facility 3, Pharmacy manager)*

Introducing robots for medication dispensing or anticancer drug preparation is also vital in redistributing pharmacy duties.

*“I believe that compounding and dispensing require a certain level of expertise, but with the use of robots and automation in those areas, there is an increasing demand for pharmacists to focus more on patient interaction.” (Facility 4, Pharmacy manager)*

As task shifting/sharing expands, pharmacists will increasingly take on roles traditionally held by physicians. Consequently, it is imperative to upgrade electronic medical record systems to ensure accountability.

*“While we are aware that having a certain level of authority comes with responsibility, it is important to note that we cannot legally assume responsibility. I believe there are some issues with the medical record system that need to be addressed in this regard.” (Facility 3, Staff pharmacist)*

### Improving professional knowledge and clinical skills

The final key factor was the pharmacists’ skills. The interview findings suggested that enhancing knowledge and experience related to cancer chemotherapy, along with interprofessional communication skills, contributes to promoting task shifting/sharing.

#### Training and updating in clinical skills.

With cancer-specialized pharmacists taking on pre-consultation interviews and delegated prescription orders, they can offer more specialized patient education and counseling.

*“Qualified pharmacists specializing in oncology can effectively utilize their expertise to provide patient counseling tailored to cancer care.” (Facility 4, Pharmacy manager)*

Standardizing workload can serve as a training tool for pharmacists and foster the professional development of all pharmacy staff.

*“I believe it is important for both physicians and proposing pharmacists to first understand the standard practices thoroughly. In clinical trials, for example, adhering to the trial protocol is essential. I’ve noticed that doctors in hospitals involved in such developments seem to have a good grasp of administration protocols. It is crucial to have this understanding because without it, there may be uncertainty about dosage adjustments. Therefore, I wanted to ensure that this knowledge is firmly embedded.” (Facility 2, Pharmacy manager)*

Through the sustained practice of task shifting/sharing, pharmacists can build trust and collaboration with physicians and other health care team members.

*“First, I think it is important to build a good relationship. I think it is important to gain trust, and after building a relationship of trust through daily pharmacist work, I would like to take things further in that direction. I think it is important to build a relationship of trust with the pharmacist. If you cannot do that, you will not be accepted (by doctors).” (Facility 1, Staff pharmacist)*

#### Creation of guidelines and procedures for pharmacy operations.

To ensure consistent quality of care regardless of pharmacists’ experience, it is important to establish common rules and systems within the pharmacy department.

*“A key issue that comes with it is how to establish rules, such as a method of entering data that ensures safety regardless of which pharmacist is performing the task, as well as how to manage the manpower and personnel allocation.” (Facility 3, Staff pharmacist)*

### Success of interprofessional collaborative practices

Finally, after overcoming barriers through these three key factors, participants who had already initiated task shifting/sharing shared their opinions on its benefits and what is needed to further promote it.

#### Effectiveness of task shifting/sharing.

Most participants from institutions that had already implemented task shifting/sharing had positive impressions. They believed the approach improved patient outcomes and safety and reduced the workloads of health care professionals.

*“Originally, we did not do it (the patient consultation about cancer chemotherapy) before the examination, but there was someone who joined halfway through. It seems like they are quite satisfied, perhaps because they talk about various things during the consultation with the pharmacist. I think there are aspects that may be difficult for patients to bring up with the doctor. Therefore, the satisfaction of being able to talk about various things may be one of the factors whether it is done before or during the examination.” (Facility 2, Staff pharmacist)*

*“The most important thing is probably ensuring safety. We are not missing any necessary tests or anything like that.” (Facility 6, Pharmacy manager)*

*“Ultimately, feedback from physicians is also important. They appreciate that protocol-based pharmacotherapy management (PBPM) helps in preemptively addressing issues that may otherwise be missed or overlooked during their short clinic hours, as well as the fact that it saves them time from having to inquire about various aspects during consultations.” (Facility 2, Pharmacy manager)*

#### Importance of assessing the effect of task shifting/sharing.

The pharmacy manager commented that to further promote task shifting/sharing, it is necessary to evaluate the efforts, present them at academic conferences, and publish them.

*“Of course, while it is important to share the task shift to alleviate physicians’ burdens, in terms of evaluating our own work, I think it is crucial for us to engage in research and publish findings, particularly as a university hospital. If we can contribute to changing healthcare reimbursements in the society through our research and reports, that would be ideal.” (Facility 5, Pharmacy manager)*

## Discussion

### Key findings

**Fig 1:**
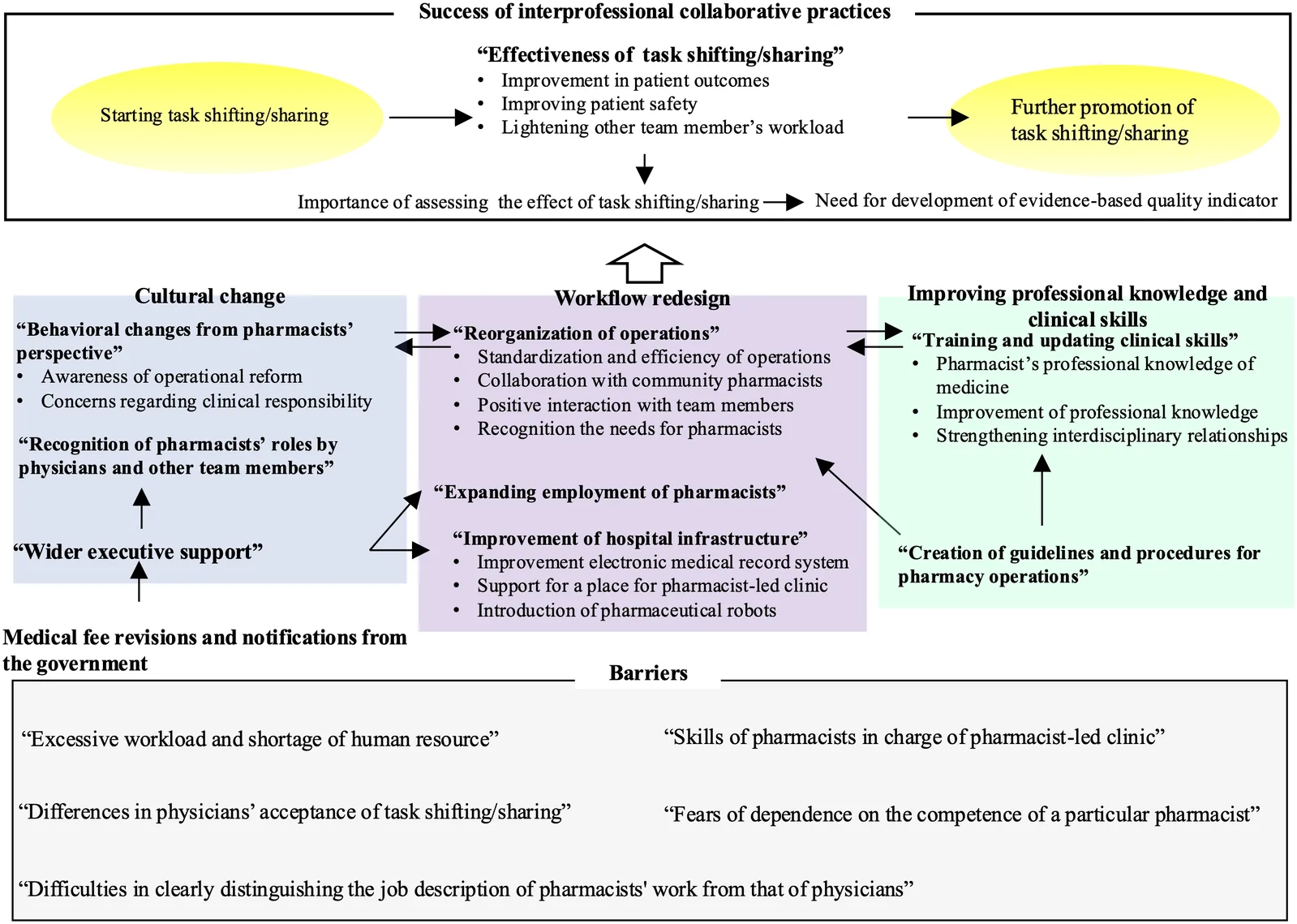
Framework for promoting task shifting/sharing from physicians to pharmacists in cancer chemotherapy in Japan.

This interview highlighted the process of implementing task shifting/sharing in oncology— specifically, its barriers to introduction and strategies for overcoming them, its effectiveness, and future expectations. Our findings suggest that task shifting/sharing was initiated through collaboration with physicians and other health care professionals, facilitated by medical fee revisions and official administrative notifications. Initially, several barriers emerged: differing attitudes toward task shifting/sharing, increased workloads for pharmacists, and challenges in ensuring staff competence. Overcoming these barriers required multifaceted strategies structured around three keywords: “Workflow redesign,” “Improving professional knowledge and clinical skills,” and “Cultural change.” To secure operational capacity, streamlining internal workflows through standardized protocols was essential. This standardization ensured consistent practice aligned with guidelines, facilitated the skill development of pharmacists in outpatient cancer chemotherapy, and enabled cross-departmental task sharing to mitigate individual workloads. Concurrently, external triggers, including medical fee revisions, prompted hospital executives to change their mindset, which accelerated staff recruitment and automated system integration. This executive transformation fostered an institution-wide cultural shift, reshaping physicians’ perceptions of the pharmacists’ roles and creating an environment more conducive to task shifting/sharing. Addressing these interconnected elements collectively allows task shifting/sharing to progress. Finally, evaluating implementation outcomes and disseminating their efficacy through academic presentations and publications can influence future medical fee revisions, generating a virtuous cycle of task shifting/sharing. Fig 1 illustrates the conceptual model of the process for promoting task shifting/sharing based on selective coding.

### Interpretation

The introduction of task shifting/sharing faced certain barriers. Three keywords, “Workflow redesign,” “Improving professional knowledge and clinical skills,” and “Cultural change” may help overcome these barriers.

Efficiency and standardization of operations are essential for systematizing workflows and securing dedicated time for pharmacists. Such improvements lead to better patient outcomes [26], shorter working hours [27], fewer medication errors [28], and cost reductions [29]. Recently, protocol-based pharmacotherapy management (PBPM) has been increasingly implemented in some hospitals. In Japan, the Health, Labor and Welfare Ministry issued a notification in April 2010 to encourage pharmacist involvement in the appropriate management of pharmacotherapy [11]. The notification recommends that changes in medication type, dosage, route of administration, duration of therapy, as well as laboratory test orders, be carried out in collaboration with physicians and other health care professionals based on protocols that have been developed and agreed upon in advance by pharmacists, physicians, and related staff. In this context, the introduction of PBPM in oncology has also expanded across hospitals, with growing evidence of its effectiveness [13–16]. Developing PBPMs tailored to each facility and the needs of other health care professionals contributes to the standardization and efficiency of pharmacy practice. Furthermore, by sharing PBPM not only within the hospital but also with nearby pharmacies, we can standardize operations between hospitals and pharmacies, which in turn fosters collaboration between pharmacies and contributes to community-wide coordination. Some participants noted that reducing pharmacists’ workload requires task shifting/sharing to non‑pharmacist personnel, such as pharmacy technicians. Studies in other countries have found that transferring such duties improved operational efficiency [30,31]. Similarly, one report indicated that duty transfer is progressing and could become more efficient in Japan [32]. However, Japan lacks a qualification system for non-pharmacists. Improving hospital infrastructure is also a facilitator of task shifting/sharing. Dispensing machines improve medical safety [33], but their implementation requires significant resources. Thus, hospital management approval or support from the government could further promote this.

To ensure patient safety, pharmacists performing medical procedures for physicians must maintain high professional skills. Promoting specialized cancer chemotherapy qualifications can demonstrate and improve pharmacists’ expertise. However, this raises the workload for some pharmacists, making duty standardization essential. Standardization serves as an educational tool and leads to the continual updating of clinical skills. This approach helps ensure the quality of care and improves patient safety, regardless of pharmacists’ experience. Clinical pathways are one method of standardizing treatment policies. Their introduction promotes multidisciplinary collaboration in cancer treatment [34]. These pathways also served as educational tools, improving patient outcomes, promoting interprofessional collaboration [35], and building trust among professionals. In addition, PBPM contributes to the standardization of pharmacists’ practice and can therefore serve as an educational tool. Establishing clinical procedures for pharmacy operations is important for duty stabilization and promoting task shifting/sharing. However, the lack of established operational rules remains a barrier.

Positive attitudes toward multidisciplinary collaboration in cancer chemotherapy are also facilitators of task shifting/sharing. In facilities where task shifting/sharing has been implemented, the cooperation of hospital directors and physicians can be an important starting point. If the hospital management approves task shifting/sharing, it will become easier for physicians and other professionals to cooperate, and other health care professionals’ perceptions of pharmacists may also become more positive. However, to faster more positive awareness of pharmacists’ roles among other medical professionals and governmental officials, it is necessary to raise awareness of the role of pharmacists among physicians and other health care professionals. To achieve this, it is important to disseminate evidence of the effectiveness of pharmacists’ contributions.

### Further intervention

The interviews highlighted barriers to task shifting/sharing. Previous studies have highlighted similar barriers including staff shortages, excessive workload, and anxiety about knowledge and skills [36–39]. From the perspective of implementation science, these findings can be comprehensively structured across individual, interprofessional, organizational, and policy levels.

At the individual level, our findings highlight that limited specialized knowledge and the pressure of increased responsibility are primary psychological barriers for staff pharmacists. Implementing a clinical pathway or PBPM addresses these individual clinical concerns by providing a structured, evidence-based protocol that guides clinical decision-making, thereby ensuring patient safety and facilitating oncology-specific skill development. Once individual competency is supported, PBPM acts as a powerful catalyst at the interprofessional level. As pharmacists demonstrate their expertise through PBPM, their clinical contributions gain wider recognition, establishing and reinforcing trusting relationships with physicians and nurses. These individual and collaborative advancements are directly sustained by the organizational level. To embed task shifting/sharing into routine hospital workflows, pharmacy managers must take proactive steps to streamline workflows and reduce the burden on staff pharmacists. This includes, for example, delegating some duties to non-pharmacist personnel and optimizing pharmaceutical operational systems.

A mindset transformation among hospital executives is vital to secure the resources necessary for long-term implementation. However, individual pharmacists or hospitals cannot overcome these barriers alone [40]. Administrative support is important [41]. At the policy level, the WHO emphasizes that governments, professional councils, and associations should work together to develop appropriate task-sharing models and interprofessional collaboration, and ensure that all cadres with a clinical role, beyond dentists, midwives, nurses, pharmacists, and physicians, also benefit systematically from accreditation and regulation processes. Specifically, support is needed to establish educational systems targeting beginners and those seeking to obtain qualifications and create environments where professionals can collaborate [42]. In Japan, while the introduction of new reimbursement incentives for clinical pharmacist activities in cancer chemotherapy has promoted task shifting/sharing, individual hospital efforts alone are not enough. Broader promotion requires vital support from government and professional organizations, including the creation of pharmacist training systems, development of guidelines and standard operating procedures, increased staffing, and facilitating access to new equipment

### Further research

As highlighted in this study, task shifting/sharing from physicians to pharmacists could improve patient outcomes and medical safety. Reporting efforts could gain administrative support and promote adoption. While studies show the effectiveness of this approach [13–16], results are difficult to generalize due to varying evaluation metrics. To promote the initiative, standardized clinical indicators across facilities are needed.

### Strengths and Limitations

This study has several strengths. Pharmacists with diverse professional backgrounds and varying years of experience were recruited, enabling the identification of challenges and key factors related to task shifting/sharing from multiple perspectives. In addition, separating discussion groups into managerial and frontline pharmacists helped minimize potential response bias associated with differences in professional roles. Importantly, the results demonstrate that although differences in clinical experience existed between managers and frontline pharmacists, their impressions and concerns regarding the promotion of task shifting/ sharing were similar.

This study has several limitations. The participating facilities were limited to large hospitals; therefore, challenges and potential solutions specific to small- and medium-sized hospitals may not have been captured. A prior study demonstrated that these hospitals perform fewer aseptic preparations and drug concentration monitoring tasks than large hospitals [43]. This suggests that smaller hospitals still face broader pharmacy practice barriers. In large hospitals with established ward duties, the keywords identified in this study promoted the initiative. By contrast, small and medium-sized hospitals continue to face fundamental barriers, making it difficult to promote these initiatives based on our findings. Specific support is essential to advance these initiatives in smaller hospitals. This study did not include an objective outcome evaluation to measure the quantitative efficacy of task shifting/sharing. Although some participants were pioneering researchers who had evaluated the usefulness of this practice and published their findings in peer-reviewed journals [13–16], their participation did not provide objective evidence of its effectiveness. Further quantitative research is needed to evaluate effectiveness. Another limitation is that, while this study reflects the perspectives of hospital pharmacists, it does not include those of physicians, nurses, community pharmacists or patients. Although focusing on hospital pharmacists allowed for more context-specific insights, the findings may reflect pharmacists’ professional aspirations or role-advocacy bias. Integrating the perspectives of other health care professionals and patients would provide a more comprehensive understanding of this service and should be explored in future research.

## Conclusions

This qualitative study highlighted that while task shifting/sharing faces barriers, they can be overcome through defined facilitators of task shifting/sharing in cancer chemotherapy. In addition, most participants perceived this approach improved patient outcomes and safety and reduced the workloads of health care professionals. To further promote this initiative, it is crucial to evaluate and publish evidence of its effectiveness. Moreover, sustained support from government bodies and professional organizations is vital for broader implementation.

## Supporting information

S1 Table Representative quotations that illustrate the key concepts identified in the analysis. (DOCX)

S1 File Consolidated criteria for reporting qualitative research (COREQ): 32-item checklist. (DOCX)

S2 File Semi-structured interview guide for participants. (DOCX)
