# Supplementary material for: Factors affecting task shifting/sharing from physicians to pharmacists in cancer chemotherapy in Japan: A qualitative study
Source: PLoS One. 2026 Sep 15;21(9):e0358247. doi: 10.1371/journal.pone.0358247 (PMC13577372; doi:10.1371/journal.pone.0358247)
Supplement: Supplementary file 1 [file pone.0358247.s001.docx]

**S1 Table. Representative quotations that illustrate the key concepts identified in the analysis.**

| Categories | Sub-categories | | Notes from the participants |
| --- | --- | --- | --- |
| Barriers | Differences in physicians’ acceptance of task shifting/sharing | | *“There are still some physicians who have not fully embraced the idea, but I feel that the number of those who are reluctant to accept proposals has significantly decreased. However, there may still be a few who remain resistant to change.” (Facility 4, Staff pharmacist)* |
|  |  |  | *“Surgeons may be more willing to delegate tasks, such as writing prescriptions, to pharmacists. They often believe that their primary roles are performing surgeries and making diagnoses, allowing them to focus on these core competencies.” (Facility 5, Pharmacy manager)* |
|  |  |  | *“Since internal medicine doctors primarily focus on prescribing medications, delegating this task to pharmacists could impede the development of medical residents. As a result, progress in this area has been slow.” (Facility 1, Pharmacy manager)* |
|  | Difficulties in clearly distinguishing the job description of pharmacists from that of physicians | | *“Pharmacists often find it challenging to define clear roles and responsibilities when collaborating with physicians or discussing task division among different professions. This ambiguity can lead to uncertainty and hesitation in decision-making.” (Facility 6, Pharmacy manager)* |
|  | Excessive workload and shortage of human resources | | *“The number of pharmacists and available space are indeed significant challenges that serve as initial barriers.” (Facility 4, Pharmacy manager)* |
|  | Skills of pharmacists in charge of pharmacist-led clinics | | *“It can be challenging to adjust duties and placements when selecting individuals with a certain level of experience rather than exclusively younger staff. Additionally, coordinating tasks and responsibilities within the ward can also present difficulties.” (Facility 5, Pharmacy manager)*  *“When it comes to areas where one’s knowledge may be lacking, it can be challenging to input all prescriptions for each patient and ensure that the prescriptions align with the physician’s intentions.” (Facility 5, Staff pharmacist)* |
|  | Fears of dependence on the competence of a particular pharmacist | | *“Fortunately, we have several oncology pharmacists, and we operate the outpatient treatment center with a team of two as a form of mentorship for younger staff. However, if one person ends up shouldering all the responsibilities, it could lead to significant burdens, especially if that person suddenly needs time off or is absent. Ultimately, it is crucial to ensure a sufficiently staffed pharmacy department to prevent such situations.” (Facility 3, Staff pharmacist)* |
| Cultural change | Medical fee revisions and notifications from the government | | *“It is my understanding that the impetus for the widespread implementation of task shifting/sharing is the government’s mandate to reform physicians’ working conditions.” (Facility 6, Pharmacy manager)* |
|  | Recognition of pharmacists’ roles by physicians and other team members | | *“I believe that consistently making such proposals has led to a greater acceptance of pharmacists. Whether it is in the pharmacist outpatient clinic or during rounds, by persistently advocating for these changes, they have become more readily accepted.” (Facility 4, Staff pharmacist)* |
|  | Behavioral changes from pharmacists’ perspective | Concerns regarding clinical responsibility | *“Since taking on more responsibilities in the pharmacy department inevitably means more work and accountability, it is understandable that pharmacists have not been actively stepping forward to request additional tasks or roles.” (Facility 3, Staff pharmacist)* |
|  |  | Awareness of operational reform | *“If we could share responsibilities within the pharmacy department, considering differences in tasks and workload on different days or times, including within the central pharmacy, it would be beneficial if we could better understand each other’s roles.” （Facility 5, Staff pharmacist）* |
|  | Wider executive support | | *“I believe system modifications require financial investment, so if the hospital is willing to invest in them to the extent necessary, and if pharmacists are genuinely desired in that regard, then it truly indicates progress.” (Facility 3, Staff pharmacist)* |
| Workflow redesign | Reorganization of operations | Recognizing the needs for pharmacists | *“It may be necessary to monitor what truly matters and what the physicians’ requests are.” (Facility 1, Pharmacy manager)* |
|  |  | Collaboration with community pharmacists | *“Since we use a common monitoring instruction manual for side effects across all medical departments, I think there is a sense of shared responsibility or delegation from physicians to pharmacists regarding the explanation of side effects to patients. By using the same shared manual, it becomes easier for pharmacists to provide follow-up between outpatient visits.” (Facility 1, Pharmacy manager)* |
|  |  | Positive interaction with team member’s | *“We could consider shifting some of the pharmacist workload to nurses if there are tasks they can handle.” (Facility 1, Pharmacy manager)* |
|  |  | Standardization and efficiency of operations | *“I think we could also handle chemotherapy orders ourselves. To pave the way for this, we could work on standardizing dosage criteria and other related factors. By doing so, we could create a situation where pharmacists are empowered to place orders themselves.” (Facility 2, Pharmacy manager)* |
|  |  |  | *“Standardizing the approach to side effects and proposing its usefulness for younger pharmacists. It serves an educational purpose for them as well.” (Facility 2, Pharmacy manager)* |
|  | Expanding employment of pharmacists | | *"In order to implement task shifting/sharing, the first step is to secure the necessary personnel and ensure there is enough capacity.” (Facility 5, Staff pharmacist)* |
|  | Improvement of hospital infrastructure | Improvement electronic medical record system | *“While we are aware that having a certain level of authority comes with responsibility, it is important to note that we cannot legally assume responsibility. I believe there are some issues with the medical record system that need to be addressed in this regard.” (Facility 3, Staff pharmacist)* |
|  |  | Support for a place for pharmacist-led clinic | *“At the outpatient treatment center, the timing seemed to be favorable, as it started in 2007 when there were various advancements in cancer care at the national level. Chemotherapy treatments were transitioning from inpatient to outpatient settings. It coincided with the completion of the three wards at the outpatient treatment center. Originally, the current location was planned to be a lookout restaurant, but owing to circumstances, it was hastily converted into the outpatient treatment center. Instead of forcing it into an existing space, we were fortunate to create a new facility, which I think was fortunate in that sense.” (Facility 3, Pharmacy manager)* |
|  |  | Introduction of pharmaceutical robots | *“I believe that compounding and dispensing require a certain level of expertise, but with the use of robots and automation in those areas, there is an increasing demand for pharmacists to focus more on patient interaction.” (Facility 4, Pharmacy manager)* |
| Improving professional knowledge and clinical skills | Training and updating in clinical skills | Pharmacists’ professional knowledge of medicine | *“Qualified pharmacists specializing in oncology can effectively utilize their expertise to provide patient counseling tailored to cancer care.” (Facility 4, Pharmacy manager)* |
|  |  | Strengthening interdisciplinary relationships  Improvement of professional knowledge | *“First, I think it is important to build a good relationship. I think it is important to gain trust, and after building a relationship of trust through daily pharmacist work, I would like to take things further in that direction. I think it is important to build a relationship of trust with the pharmacist. If you cannot do that, you will not be accepted (by doctors).” (Facility 1, Staff pharmacist)* |
|  |  |  | *“I believe it is important for both physicians and proposing pharmacists to first understand the standard practices thoroughly. In clinical trials, for example, adhering to the trial protocol is essential. I’ve noticed that doctors in hospitals involved in such developments seem to have a good grasp of administration protocols. It is crucial to have this understanding because without it, there may be uncertainty about dosage adjustments. Therefore, I wanted to ensure that this knowledge is firmly embedded.” (Facility 2, Pharmacy manager)* |
|  | Creation of guidelines and procedures for pharmacy operations | | *“A key issue that comes with it is how to establish rules, such as a method of entering data that ensures safety regardless of which pharmacist is performing the task, as well as how to manage the manpower and personnel allocation.” (Facility 3, Staff pharmacist)* |
| Success of interprofessional collaborative practices | Effectiveness of task shifting/sharing | Improvement in patient outcomes | *“Originally, we did not do it (the patient consultation about cancer chemotherapy) before the examination, but there was someone who joined halfway through. It seems like they are quite satisfied, perhaps because they talk about various things during the consultation with the pharmacist. I think there are aspects that may be difficult for patients to bring up with the doctor. Therefore, the satisfaction of being able to talk about various things may be one of the factors whether it is done before or during the examination.” (Facility 2, Staff pharmacist)* |
|  |  | Improving patient safety | *“The most important thing is probably ensuring safety. We are not missing any necessary tests or anything like that.” (Facility 6, Pharmacy manager)* |
|  |  | Lightening other team member's workload | *“Ultimately, feedback from physicians is also important. They appreciate that PBPM helps in preemptively addressing issues that may otherwise be missed or overlooked during their short clinic hours, as well as the fact that it saves them time from having to inquire about various aspects during consultations.” (Facility 2, Pharmacy manager)* |
|  | Importance of assessing the effect of task shifting/sharing | | *“Of course, while it is important to share the task shift to alleviate physicians’ burdens, in terms of evaluating our own work, I think it is crucial for us to engage in research and publish findings, particularly as a university hospital. If we can contribute to changing healthcare reimbursements in the society through our research and reports, that would be ideal.” (Facility 5, Pharmacy manager)* |
