# Supplementary material for: Factors affecting task shifting/sharing from physicians to pharmacists in cancer chemotherapy in Japan: A qualitative study
Source: PLoS One. 2026 Sep 15;21(9):e0358247. doi: 10.1371/journal.pone.0358247 (PMC13577372; doi:10.1371/journal.pone.0358247)
Supplement: Supplementary file 3 [file pone.0358247.s003.docx]

S2 File. Semi-Structured interview guide for participants.

1. What do you do as “task shifting/sharing” in your hospital?

What enabled you to deliver this “task shifting/sharing”?

What would you like to do in the future?

2. What do you think has been the effect of “task shifting/sharing”?

What do you think will be the effect of starting a new “task shifting/sharing” in the future?

*PROMPT: How about effects on your patients*

*PROMPT: How about effects on other medical staffs*

3. What were the initial barriers of “task shifting/sharing”?

What hindered the implementation of “task shifting/sharing”? What support do you need to implement “task shifting/sharing”?

*PROMPT: What was the impression of it from other medical staff?*

*PROMPT: What was the impression of it from other pharmacists?*

4. What advice would you give to other facilities that are starting task shifting/sharing?
